# Association of Physical Activity and Quality of Life with Work-Related Musculoskeletal Disorders in the UAE Young Adults

**DOI:** 10.3390/healthcare10040625

**Published:** 2022-03-26

**Authors:** Mennatallah Adel Mohamed Mohmoud Alseminy, Baskaran Chandrasekaran, Kalyana Chakravarthy Bairapareddy

**Affiliations:** 1 Department of Physiotherapy, College of Health Sciences, University of Sharjah, Sharjah 27272, United Arab Emirates; u18106066@sharjah.ac.ae; 2 Department of Exercise Science & Sports, Manipal College of Health Professions, Manipal Academy of Higher Education, Manipal 576104, India; baskaran.c@manipal.edu

**Keywords:** musculoskeletal diseases, musculoskeletal pain, physical activity, occupational diseases, quality of life, mental health

## Abstract

Background: Work-related musculoskeletal disorders (WRMSDs) pose threat to the global economy and work productivity. Though growing evidence shows physical activity and quality of life are major determinants for WRMSDs, the association between physical activity and the quality of life among the young adults of the United Arab Emirates (UAE) remains unclear. Methods: In a cross-sectional study, a total of 507 young adults who were between the ages 18–35 years were administered an interviewer-based survey on musculoskeletal disorders, physical activity, and quality of life. The association between the potential determinants and the WRMSDs was analyzed using linear and logistic regression models. Results: High prevalence (75%) of WRMSDs was found among the UAE young adults. Participants with low leisure-time physical activity had few WRMSDs. There was no significant association between physical activity or quality-of-life scores with the incidence of WRMSDs although physical activity time was highly associated with the quality of life, especially the social domain. Conclusion: Though a high prevalence of WRMSDs among UAE men and women was found, neither physical activity nor the quality-of-life scores determined the incidence of WRMSDs.

## 1. Introduction

In spite of technological advances, many work sectors require workers to perform their tasks repeatedly or for a long duration in a single posture, resulting in work-related musculoskeletal disorders (WRMDs). WRMDs are widely recognized as a significant cause of the pain and disability among sedentary workers [1].

### 1.1. Impact of Work-Related Musculoskeletal Disorders

WRMSDs encompass the wider range of non-traumatic injuries or dysfunction associated with muscles, tendons, ligaments, nerves, cartilages, spinal discs, and joints of upper limb, neck, and low back [1,2]. Neck pain and low back pain are the highly prevalent WRMSDs encountered in primary care. The direct and indirect healthcare costs of WRMSDs in terms of sick leave and work productivity are estimated to be €2753 in Sweden, while in the Netherlands, low back pain is estimated to cost 0.9% (€2753 million) of total health care costs [3,4].

### 1.2. Potential Determinants of WRMSDs

WRMSDs are multifactorial with risk factors ranging from non-modifiable risk factors such as age and gender to modifiable risk factors such as altered biomechanics during work (lifting, pulling and pushing, repetition), physical fitness, and psychosocial factors (judgement, memory, and boredom) [5].

#### 1.2.1. Physical Activity and Its Effect on WRMSDs

Epidemiological studies have attempted to establish a bidirectional association between physical activity levels and WRMSDs [6,7]. Moderate physical fitness and physical activity levels were found to have a propensity towards fewer WRMSDs. Higher leisure time physical activity levels resulting in improved muscular endurance, joint integrity, and motor control are postulated to offer protection against 5h3 incidence of WRMSDs [6]. Conversely, high work-related physical activity levels are also speculated to result in physical, mental fatigue, and cognitive dysfunction, which may increase the WRMSD risk [8,9]. Hence, the empirical evidence demonstrating the inverse association between physical activity and WRMDs remains mixed.

#### 1.2.2. Quality of Life (QOL) and WRMSDs

The World Health Organization (WHO) defines QOL as “an individual’s perception of their position in life in the context of the culture and value systems in which they live and in relation to their goals, expectations, standards and concerns” [10]. Thus, QOL indicates the physical, social, and psychological wellbeing of an individual while maintaining an ideal interaction and balance between oneself and the environment. Observational and longitudinal studies have established an inverse association between the quality of life and WRMSDs [11,12,13]. WHO developed the “WHO Quality of Life Scale Brief Version” (WHOQOL-BREF) to facilitate the assessment of four domains, physical, psychological, social, and environment, that determine the quality of life in young adults [14]. However, the existing evidence regarding the prevalence of the WRMSDs and the risk factors remains mixed.

### 1.3. Industrialization and Cross-Cultural Factors of WRMSDs

Due to industrialization and westernization, United Arab Emirates (UAE), the third most populous Arab country, is becoming a fast-growing hub for workforces [15]. Information and communication technology sectors are becoming one of the UAE’s rapidly developing and strongest economic sectors providing opportunities for more jobs, productivity, nation impact, and growth [15]. While skilled workforce opportunities are rising in the UAE, so do the WRMSDs which are identified as the “common causes for disability and limitation related to daily living and gainful employment” [16]. In their recent narrative synthesis, Shaikh and colleagues (2020) found a significant rise in the prevalence of WRMSDs ranging from 11–34% among healthcare workers including nurses and dentists in the United Arab Emirates [16]. However, knowledge of the intensity of WRMSDs problems and their potential determinants such as physical activity and quality of life among other skilled professional workers (information and communication technology) in UAE young adults is still lacking.

### 1.4. Aim of the Study

We aimed to administer a cross-sectional survey with two objectives: (1) to establish the prevalence of WRMSDs among working UAE adults; (2) to demonstrate whether an association exists between physical activity and quality of life with WRMSDs in Arab adults. The prevalence and the strength of the association between the proposed risk factors (physical activity or fitness and self-reported quality of life) may help public health experts and behavioral scientists to develop or design appropriate cross-cultural intervention for risk factor modification in the long-term prevention and management of WRMSDs in the Arab population.

## 2. Subjects and Methods

### 2.1. Study Design and Setting

The present cross-sectional study was administered among people dwelling in and around Sharjah between 15 September 2020 and 21 January 2021. The study was reported as per the STrengthening the Reporting of OBservational studies in Epidemiology (STROBE) guidelines Supplementary Table S1 and complied with the tenets of the Declaration of Helsinki. The study was conducted with the approval of the ethical committee of the University of Sharjah (REC-20-04-23-01-S). The volunteered participants provided necessary informed and written consent.

### 2.2. Participants

The potential participants eligible to participate in the study were adults, aged >18 years and <60 years, working in software and technology firms in and around Sharjah. Further, the participants had to have at least one-year experience in the current job and know Arabic or English. Potential participants who had self-reported cardiovascular disorders or musculoskeletal disorders that limited their participation in physical activity were excluded from the study. Further, participants with established depressive or anxiety disorders, which were perceived to affect the quality of life of the workers, were excluded from the study.

### 2.3. Outcome Variables and Measurement

As the study aimed to address the association of physical activity levels and perceived quality-of-life scores with musculoskeletal pain, we used a self-reported questionnaire containing four parts: (1) Participant demographics, (2) Self-reported physical activity levels using the Global Physical Activity Questionnaire (GPAQ), (3) Quality-of-life evaluation using the WHO quality-of-life short form (WHO BREF) scale, and (4) musculoskeletal pain scores using the Nordic musculoskeletal questionnaire (NMQ).

(1) Patient demographics: This part of the questionnaire asked about age, gender, years of experience, nature of work, and family history of musculoskeletal disorders (rheumatoid arthritis or spondyloarthropathies)
(2) Self-reported physical activity levels: WHO developed the Global Physical Activity Questionnaire (GPAQ) for surveillance of physical activity across countries. The GPAQ consists of 16 questions on physical activity based on three domains: activity at work, commuting, and recreational activities. The GPAQ evaluates the three dimensions of physical activity: frequency, duration, and intensity. The criterion validity of the GPAQ with accelerometer-derived physical activity is fair (r = 0.23–0.26) in high-income countries [17].
(3) WHO Quality-of-life scores: Quality of life was assessed using the WHOQOL-BREF, which consists of 26 questions, including physical, psychological, social, and environmental domains [18]. The response options ranged from 1 (very dissatisfied/very poor) to 5 (very satisfied/very good). The final scores of overall QOL (ranging 16–80) and of each domain (ranging 4–20) were calculated according to the developers. A higher score indicated a better perception of life quality. A Norwegian validation study found an acceptable convergent and discriminate validity and internal consistency across physical, psychological, and environmental domains, but the social domain showed only marginal reliability [19].
(4) *Musculoskeletal pain:* Musculoskeletal discomfort was assessed using the NMQ, which is widely used in recent Arabian epidemiological studies [20,21]. The Nordic musculoskeletal questionnaire asks about overall and site-specific musculoskeletal problems for seven days over a 12-month period in the first section while the following section assesses the impact of the musculoskeletal problems on an individual’s capacity to work as well as their overall quality of life in the workplace. To assist participants in answering questions focusing on musculoskeletal symptoms, an anatomical diagram was used to clearly identify body regions. The anatomical diagram and the relevant questions cover nine body regions, namely, the neck, shoulders, elbows, wrists/hands, upper back, lower back, hips/thighs/buttocks, knees, and ankles). The NMSQ was found to have marginal validity and reliability (0–23%) [22].

### 2.4. Bias

As factors such as diet, socioeconomic status, and environmental factors such as availability of exercise equipment, organization structure, and other individual factors have an effect, we framed the survey to include the above factors. The selection bias was inevitable as the volunteers were chosen in and around the city of Sharjah

### 2.5. Sample Size

To show a prevalence of 30% of WRMSDs at a precision of 5%, we required 506 samples at a significance of 95% [16]. With an estimated proportion of 0.3 and allocation ratio of 0.03 with 90% power and 95% significance, a sample size of 506 was estimated using G*Power software (University of Kiel, Kiel, Germany).

### 2.6. Procedure

The study and its whereabouts were advertised on noticeboards of hospitals, community centers, and the residential areas in and around a multispecialty university. The recruitment was advertised on the social media pages of the investigators. The volunteers who contacted the primary investigator, with self-reported WRMD, were later confirmed by an orthopedic physician of a multispecialty university teaching hospital. The diagnoses of WMSDs were where the diagnostic criteria were fulfilled according to the European guidelines from the European Union Information Agency for Occupational Safety and Health [23]. Sites of WMSDs among the participants were obtained and grouped into three categories: upper back, lower back/sciatica, and extremities.

The survey questionnaire was primarily developed in English and later was translated into Arabic by a native Arabic expert. Then, the translated questions were back-validated in English. The intraclass correlation coefficient (ICC) for the translated version of the Arabic questionnaire ranged from 0.765 to 0.928, with excellent correlation (ICC  =  0.824). Cronbach’s alpha was 0.792 for the Arabic version of the survey questionnaire, indicating good internal consistency. The primary investigator administered the questionnaire to the volunteers in person. Once the data were obtained from the participants, the necessary data regarding the physical activity levels, quality-of-life scores, and the musculoskeletal problems (intensity, site, frequency, and duration) were sought and compiled for data analysis.

### 2.7. Statistical Analysis

As the data were not normally distributed, as shown by the Shapiro Wilk test (*p* < 0.05), the physical activity, quality-of-life scores, and musculoskeletal discomfort were log transformed until normality was met. Descriptive data are presented as means and standard deviations (SD) for continuous variables and as *n* (%) for categorical variables. Gender differences regarding physical activity, quality of life, and musculoskeletal pain were analyzed using the Mann Whitney U test. The prevalence was calculated as the number of participants with the presence of musculoskeletal pain for each body area out of the total number of participants included in the study. Associations between the musculoskeletal pain (dependent, categorical variable) and the physical activity dimensions (predictors) were analyzed using logistic regression analysis using the enter method and generalized estimating equations while linear regression was used to relate the quality-of-life scores (dependent, continuous variable) and the physical health dimensions (predictors). The relationships between the physical activity dimension and the quality-of-life domains were illustrated by scatter plots. Furthermore, regression plots were drawn to show the odds of not having a musculoskeletal disorder associated with each of the physical activity dimensions (frequency, duration, and intensity). The significance level was set to *p* < 0.05. All statistical analyses were carried out using the statistical software JASP version 0.14.1.0 (JASP team, 2020, University of Amsterdam, Amsterdam, The Netherlands)

## 3. Results

### 3.1. Participants

A total of 507 participants completed the questionnaire. As in one case, data were incomplete, only the completed data from 506 participants were analyzed and presented. The study included a higher number of male participants (63%) compared to female counterparts (37%). Table 1 shows the baseline characteristics of the participants who completed the survey. The bottom of Table 1 presents the prevalence of the WRMSDs among the participants.

### 3.2. Descriptive Data

The reported frequency of lower back, neck, and shoulder pain was relatively high. Pain in the upper back, knee, and hand was the other common work-related musculoskeletal condition with moderate prevalence between 20 and 40%. Hip pain and elbow pain were less prevalent compared to other body regions with a low prevalence of 15% and 9%, respectively. The prevalence of work-related musculoskeletal conditions was found to be relatively high in young adults of the UAE.

Table 2 shows the differences in physical activity variables based on gender and WRMSDs. The physical activity levels were significantly lower among all participants. The work time physical activity levels were relatively low in females compared to male participants (*p* = 0.42), whereas there was no statistically significant difference in the other domains and the total physical activity levels. There was no statistically significant difference in total PA levels and PA domains among young adults of the UAE.

The active transport domain PA levels were lower in participants with neck pain compared to those who did not report neck pain (*p* = 0.04). The comparison of other domains and total scores of PA between the participants with neck pain and those without neck pain was not statistically significant (*p* > 0.05). The comparison of PA domains and the total PA scores was not statistically significant in shoulder and upper back areas of the Nordic questionnaire (*p* > 0.05). The comparison of PA domains and scores was not statistically significant in participants who reported wrist pain, lower back, and hip pain compared to those who did not report WRMSD pain in the same regions (*p* > 0.05). The worktime PA levels were high and statistically significant in participants with knee pain compared to those who did not report knee pain (*p* = 0.05). The work domain physical activities and daily sitting time were significantly lower (*p* = 0.04 and 0.02, respectively) in participants who reported neck pain (Table 3)

### 3.3. Outcome Data

Table 3 shows the differences in the quality-of-life scores based on physical activity and gender differences. Our study found no significant differences in the quality-of-life scores among the participants with or without WRMSDs. However, there was a significant difference in quality-of-life scores, especially social relationships, when stratified across age with younger participants scoring poorly in social relationship compared to older participants

Table 4 shows the relationship between the physical activity dimensions and the quality-of-life domains. Sedentary time was inversely associated (−0.126, *p* < 0.01) with the physical domain scores but positively related to the psychological domain scores (−0.091, *p* < 0.05). Physical activity time was positively related to the social domain scores (−0.104, *p* < 0.05)

### 3.4. Main Results

The study determined the correlation between the physical activity levels and health-related quality-of-life domains according to each body area. There was no statistically significant correlation between PA values and health-related quality-of-life domains in the study population.

**Figure 1:**
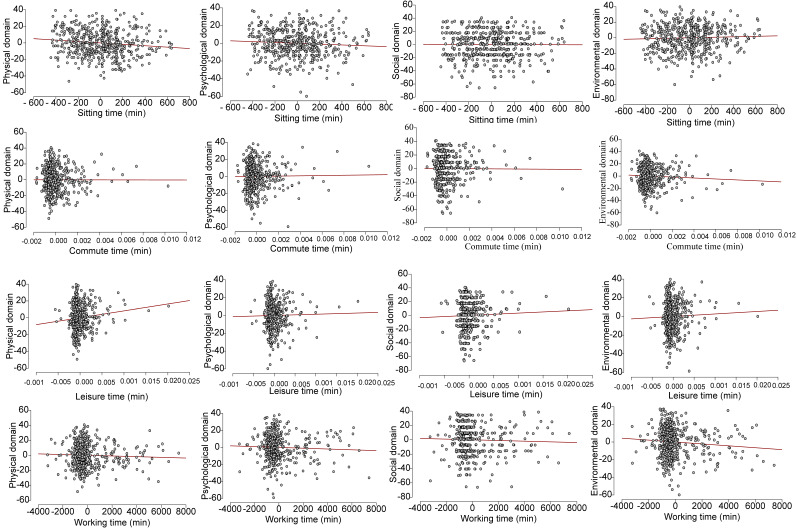
Scatter plots showing the relationship between the sedentary and physical activity dimensions (commute, leisure, and work) and quality-of-life domains.

**Figure 2:**
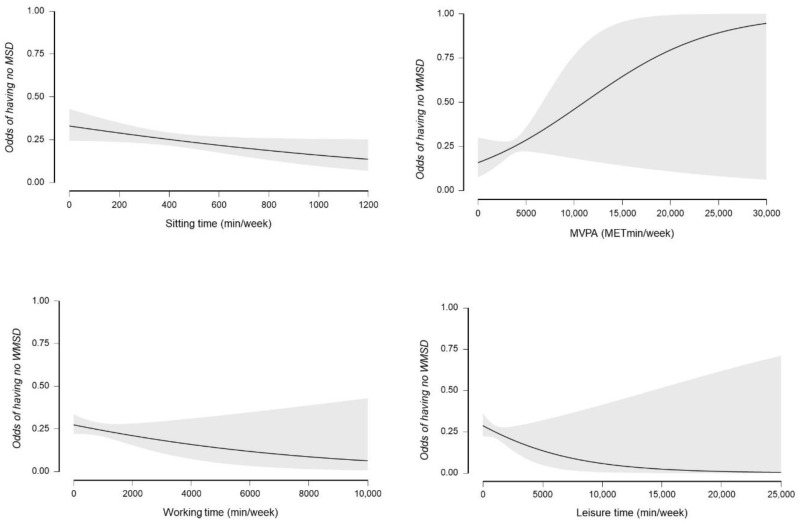
Association between the odds of not having WRMSDs and the physical activity variables. Except for MVPA (METmin/week), all other variables (sitting time, leisure time high intensity exercise, and working time) were inversely associated with the probability of not having WRMSDs; Abbreviation: MVPA = Moderate–vigorous physical activity.

In regression analysis, sitting was negatively associated with musculoskeletal disorders and the quality-of-life scores. Though overall physical activity was not associated with the musculoskeletal disorders, each individual physical activity dimension [sitting time (β = 1.316, *p* < 0.001), work (β = 0.498, *p* < 0.001), leisure (β = 0.316, *p* < 0.001), commute (β = 0.412, *p* < 0.001), and sitting time (β = 1.433, *p* < 0.001)] was independently and positively associated with the quality-of-life scores (Table 5). Figure 1 illustrates the relationship between the quality-of-life scores and the physical activity dimensions, while Figure 2 demonstrates the odds of having no musculoskeletal disorder with each of the physical activity domains. As depicted in Figure 1, our study found a non-significant relationship between individual domains of the quality-of-life scores and physical activity (r = −0. 34 to 0.68; *p* = 0.162)

## 4. Discussion

Our study aimed to explore the prevalence and determinant of WRMSDs in the young adults of the UAE. We found significant prevalence of WRMSDs among the UAE working population (75%). We also found that physical activity and quality of life were found to favorably affect the incidence of WRMSDs

### 4.1. Prevalence of WRMSDs

We found a high prevalence of overall WRMSDs (75%) among the UAE population, with low back pain being the most common WRMSDs followed by neck, shoulders, and upper back and then elbows, hips, and thighs. This may be probably due to young respondents in our survey, who were working in information and communication technology sectors for extended hours in a constant posture. The reported prevalence in our study is higher than the prevalence reported in the review findings of Shaikh and colleagues (2020), who reviewed WRMSDs and the risk factors in Emirati dentists and nurses [16]. However, Shaikh and colleagues (2020) did not review the WRMSDs among the Emirati working in information and communication technology sectors, whereas our study involved young computer professionals of the UAE. Our findings concur with observational studies that claim a high prevalence of musculoskeletal pain and loss of work productivity immediately after computer work [24,25]. Poor prolonged posture, prolonged activation of the small muscles (hand muscles) during low-level contraction such as typing, and inappropriate ergonomically designed workstations while using computers or laptops are plausible mechanistic links to musculoskeletal problems, mainly back pain, neck pain, headache, and wrist pain [24,26]. However, we found lower prevalence of wrist and hip pain probably due to the younger respondents of our study.

### 4.2. Determinants of WRMSDs among the UAE Population

The participants with no WRMSDs had lower incidental and intentional activities such as work time activity, active transport, and leisure time activities. WRMSD prevalence was low in participants with a shorter daily sitting time and moderate–vigorous physical activities or total PA volume. However, the differences did not reach statistical significance. Our study findings concur with the existing observational studies that have established a significant relationship between high occupational physical activity and the incidence of WRMSDs [27,28,29]. A high level of occupational physical activity may lead to poor sustained posture, repetitive stress to the bones, joints, and ligaments, and physical and mental fatigue that may end in significant musculoskeletal pain, poor work productivity, and quality of life.

Conversely, our study findings that participants with low leisure time physical activity had low WRMSDs are worth pondering as these findings contradict results of existing epidemiological studies claiming the musculoskeletal benefits of leisure physical activity [30,31]. Leisure time sports were found to favor physical fitness and low stress as well as sickness absenteeism [32]. However, we did not observe a significant relationship probably due to the cross-cultural differences and the climatic variation for outdoor leisure time in the UAE. We recommend that future empirical studies address this non-significant relationship between the leisure time physical activity and WRMDs.

Similarly, we did not find any significant differences between the participants with or without WRMSDs across the four quality-of-life score domains: physical, psychological, social, and environmental domains. Our findings contradict the previous epidemiological findings that established a bidirectional association between the quality of life and WRMSDs. A high incidence of WRMSDs was observed among the individuals who had a low quality of life, and a high incidence of WRMSDs results in a poor quality of life [11,33]. Those who perceive better work ability without pain may have a good self-reported quality of life whereas those who have a good quality of life may perceive less musculoskeletal discomfort during work [11]. This non-significance may be due to the inclusion of younger participants who have lesser experience and a good socioeconomic status, which might have rendered the relationship non-significant.

### 4.3. Association between Physical Activity, Quality of Life, and WRMSDs

While we did not find any association between physical activity and WRMSDs, total physical activity time improved the quality-of-life scores. Unsurprisingly, sedentary time was associated with lower WRMSD incidence in our study. We postulate that intermittent rest periods (rest microbreaks) may reduce occupation-related musculoskeletal injuries. Rest microbreaks reduce repetition and monotony that might reduce the risk of WRMSDs in workplaces [34,35]. Recently, active microbreaks (not related to occupational physical activity) were found to reduce WRMSDs [36]. High occupational physical activity is associated with increased physical and psychological dysfunction and increased propensity to WRMSDs [30,37]. Further, sedentary time was inversely associated with the physical domain of quality-of-life scores but favorably associated with psychological domains. Excessive sedentary behavior and high physical inactivity are associated with early cardiometabolic risk and adverse psychological effects. However, our study failed to establish a relationship between sedentary time and WRMSDs.

While overall physical activity time improves social domain measures, we did not find any significant relationship between physical activity and the environmental domains of the quality-of-life scores. Physical activity is well-known for its social influence with improved social interactions and improved intercommunity transition and communication [38,39]. However, information related to the environmental aspects of physical activity is still unclear.

### 4.4. Practical Applications of Our Findings

There is a huge Arab work force comprising young adults working in software and information technology fields, and there is a dire need to implement necessary strategies to minimize WRMSDs. As quality of life and WRMSDs are bidirectional, public experts and behavioral scientists should design public policies that can minimize WRMSDs with physical activity and implement quality-of-life improvement strategies in the young Arab working population.

### 4.5. Study Limitations

Although we established the prevalence and the determinants of WRMSDs among young adults of Sharjah, the present study has several limitations: (1) as a cross-sectional study, the causal relationship of the WRMSDs with physical activity and quality of life cannot be inferred. We recommend that readers be cautious while using physical activity or quality-of-life management for WRMSD management or prevention [40]; (2) we targeted people randomly in the society; however, only young adults responded to our survey. There is definitely a huge number of young adults who suffer from non-specific musculoskeletal pain in the industries and factories that did not participate in the study as we did not target a specific place; (3) the administered questionnaire was self-reported, with drawbacks of social desirability or reactivity bias and recall bias. We recommend that future studies measure physical activity objectively to make the findings more valid [41].

## 5. Conclusions

Work-related musculoskeletal disorders were found to be extremely high among the UAE population. Although a long physical activity time was associated with improved quality of life, the relationship with work-related musculoskeletal problems remains unclear. High occupational physical activity was found to increase the WRMSD risk in the workplace and impair the quality of life among UAE adults. We urge public health experts and national governance to incorporate behavioral strategies to limit sedentary behavior, incorporate work–break schedule, and improve leisure time opportunities among young computer professionals in the UAE to reduce WRMSDs.

## Figures and Tables

**Table 1:** Descriptive characteristics of the participants.

Baseline Characteristics		*n* (%)
Gender	Male	319 (63.0)
	Females	187 (37.0)
Age group	18–29	287 (56.7)
	30–39	219 (43.3)
Work-related musculoskeletal disorders ^#^	Presence of any one of WMSDs	379 (74.75)
	Neck	201 (40)
	Shoulders	207 (41)
	Upper back	164 (32)
	Elbows	47 (9)
	Wrists/hands	99 (20)
	Lower back	244 (48)
	Hips/Thighs	77 (15)
	Knees	142 (28)
	Ankles/Feet	103 (20)

^#^ Presence assessed using the Nordic questionnaire; Abbreviation: WMSD = Work-related musculoskeletal disorder (assessed using the Nordic Questionnaire).

**Table 2:** Results of the GPAQ domains by gender and musculoskeletal conditions.

Variables		Work Time Activity ^#^ (min/week)	Active Transport ^#^ (min/week)	Leisure Time Activities ^#^ (min/week)	Daily Sitting Time ^#^ (min/week)	Moderate Intensity Activity ^#^ (min/week)	Vigorous Activities ^#^ (min/week)	MVPA ^#^ (min/week)	Total PA ^#^ (min/week)
Gender	Male	1792.20 ± 3515.26	680.44 ± 1229.95	1515.92 ± 2411.38	421.91 ± 223.44	1345.32 ± 1867.43	1962.80 ± 3030.81	3988.55 ± 4546.15	751.79 ± 823.16
	Female	1108.42 ± 2208.44	668.60 ± 1343.57	1693.14 ± 2967.35	459.81 ± 244.82	1312.84 ± 1927.64	1488.72 ± 2869.36	3470.17 ± 4322.40	681.45 ± 820.20
	*W*	30,658.00	29,817.50	29,556.50	30,658.00	30,716.00	28,267.00	30,888.50	30,657.00
	*p* value	0.042 *	0.931	0.530	0.128	0.873	0.140	0.282	0.427
Age Group	18–29	1466.61 ± 2964.80	635.44 ± 1252.02	1368.25 ± 2063.87	452.94 ± 243.33	1248.76 ± 1781.86	1586.10 ± 2553.91	3470.299 ± 4006.62	669.31 ± 749.72
	30–39	1672.03 ± 3361.39	733.418 ± 1294.02	1871.65 ± 3221.30	410.41 ± 212.27	1453.04 ± 2023.13	2090.63 ± 3477.24	4277.09 ± 5023.05	807.94 ± 909.29
	*W*	26,253.00	28,263.50	26,243.00	24,233.00	27,238.00	25,322.00	29,239.00	23,152.50
	*p* value	0.530	0.459	0.065	0.078	0.299	0.104	0.083	0.105
WRMSDs	Yes	832.24 ± 1643.96	676.29 ± 1269.22	501.68 ± 935.13	435.21 ± 231.55	1076.43 ± 2135.39	720.00 ± 2073.11	3806.64 ± 4470.04	727.11 ± 821.72
	No	762.21 ± 1653.39	675.43 ± 1025.79	623.31 ± 989.90	392.66 ± 232.93	1028.35 ± 1827.39	452.28 ± 1302.63	3541.58 ± 4369.97	700.32 ± 812.70
	*W*	25,323.00	22,713.50	22,633.50	26,330.00	25,325.50	24,522.00	24,583.00	24,266.00
	*p* value	0.172	0.995	0.212	0.074	0.678	0.820	0.561	0.750

^#^ Expressed as the mean ± standard deviation; * level of significance revealed by the Mann Whitney U test; Abbreviations: MVPA = Moderate–vigorous physical activity; PA = Physical activity; WRMSDs = Work-related musculoskeletal disorders; W = Mann Whitney U statistic.

**Table 3:** Results of the QoL domains based on gender and musculoskeletal conditions.

Baseline Variables		Quality of Life ^$^			
		Physical Health ^#^	Psychological Domain ^#^	Social Relationships ^#^	Environmental Domain ^#^
Gender	Male	61.27 ± 15.68	63.38 ± 15.17	64.43 ± 20.33	66.11 ± 16.57
	Female	62.41 ± 16.02	65.29 ± 14.54	66.29 ± 19.94	65.01 ± 17.31
	W	26,855.00	28,290.50	28,931.50	28,032.50
	*p* value	0.503	0.237	0.393	0.546
Age	18–29	60.84 ± 15.96	63.29 ± 15.56	63.01 ± 20.92	65.61 ± 17.33
	30–39	62.82 ± 15.53	65.11 ± 14.057	67.99 ± 18.80	65.88 ± 16.14
	W	26,834.00	27,393.50	26,793.50	28,3452.50
	*p* value	0.231	0.244	0.018 *	0.877
WRMSDs	Yes	61.67 ± 15.79	64.05 ± 14.96	65.08 ± 20.19	65.73 ± 16.82
	No	61.19 ± 15.62	64.67 ± 16.23	65.68 ± 21.09	64.79 ± 16.73
	W	24,840.00	23,849.50	23,603.00	24,950.00
	*p* value	0.587	0.685	0.743	0.535

^#^ Expressed as the mean ± standard deviation; W = Mann Whitney U statistic; * level of significance revealed by the Mann Whitney U test; ^$^ derived from WHOQOL BREF scores; Abbreviations: WRMSDs = Work-related musculoskeletal disorders.

**Table 4:** Correlation between physical activity (measured with GPAQ) and quality of life (measured with WHOQoL-BREF) domains.

Physical Activity Dimensions (min/week)	Quality of Life Domains							
	Physical		Psychological Domain		Social		Environmental	
	r	*p*	r	*p*	r	*p*	r	*p*
MVPA	0.011	0.813	0.009	0.833	−0.104	0.019 *	−0.040	0.373
Total PA	0.005	0.903	0.22	0.628	−0.104	0.020 *	−0.024	0.586
Sedentary time	−0.128	0.004 **	−0.091	0.041 *	0.003	0.942	0.059	0.188

** Correlation is significant at the 0.01 level, * Correlation is significant at the 0.05 level (2-tailed), r = correlation coefficient; p significance (2-tailed), Abbreviations: MVPA moderate to vigorous physical activity; PA physical activity.

**Table 5:** Association between the dimensions of physical activity with the quality-of-life scores and work-related musculoskeletal disorders.

Independent Variables		Physical Activity (Predictor)	β	SE	*p*
Musculoskeletal disorders (WMSD)		Sitting time	−0.001	0.000	0.043 *
		Work—vigorous intensity	−0.000	0.000	0.058
		Work—moderate intensity	−0.000	0.000	0.193
		Leisure—vigorous intensity	−0.000	0.000	0.174
		Commute	−0.000	0.000	0.246
		MVPA	0.000	0.000	0.164
Quality of Life Domains (WHOQOL)	Physical	Sitting time	1.316	0.003	<0.001 **
		Work—vigorous intensity	0.100	0.001	0.296
		Work—moderate intensity	0.498	0.001	<0.001 **
		Leisure—vigorous intensity	0.285	0.001	0.002 *
		Leisure—moderate intensity	0.316	0.002	<0.001 **
		Commute	0.412	0.001	<0.001 **
	Psychological	Sitting time	1.433	0.003	<0.001 **
		Work—vigorous intensity	0.156	0.001	0.117
		Work—moderate intensity	0.520	0.001	<0.001 **
		Leisure—vigorous intensity	0.386	0.001	<0.001 **
		Leisure—moderate intensity	0.222	0.002	0.020 *
		Commute	0.445	0.001	<0.001 **
	Social	Sitting time	1.130	0.004	<0.001 **
		Work—vigorous intensity	0.016	0.001	0.843
		Work—moderate intensity	0.392	0.001	<0.001 **
		Leisure—vigorous intensity	0.227	0.001	0.004 **
		Leisure—moderate intensity	0.191	0.002	0.015 *
		Commute	0.319	0.001	<0.001 **
	Environmental	Sitting time	1.378	0.003	<0.001 **
		Work—vigorous intensity	0.157	0.001	0.084
		Work—moderate intensity	0.400	0.001	<0.001 **
		Leisure—vigorous intensity	0.398	0.001	<0.001 **
		Leisure—moderate intensity	0.216	0.002	0.014 *
		Commute	0.326	0.001	<0.001 **

** Correlation is significant at the 0.01 level, * Correlation is significant at the 0.05 level (2-tailed), p significance (2-tailed), Abbreviations: MVPA = moderate to vigorous physical activity, WHOQOL = World Health Organization Quality of Life questionnaire.

## Data Availability

The data that support the findings of this study are available from the corresponding author, [KCB], upon reasonable request.

## Supplementary Materials

The following supporting information can be downloaded at: https://www.mdpi.com/article/10.3390/healthcare10040625/s1, Table S1: STROBE Statement for cross sectional studies.
